# The production of a physiological puzzle: how *Cytisus adami* confused and inspired a century’s botanists, gardeners, and evolutionists

**DOI:** 10.1007/s40656-018-0207-0

**Published:** 2018-08-21

**Authors:** John Lidwell-Durnin

**Affiliations:** London, UK

**Keywords:** Heredity, Horticulture, Botany, Periodicals, Darwin, Pangenesis

## Abstract

‘Adam’s laburnum’ (or *Cytisus adami)*, produced by accident in 1825 by Jean-Louis Adam, a nurseryman in Vitry, became a commercial success within the plant trade for its striking mix of yellow and purple flowers. After it came to the attention of members of *La Société d’Horticulture de Paris,* the tree gained enormous fame as a potential instance of the much sought-after ‘graft hybrid’, a hypothetical idea that by grafting one plant onto another, a mixture of the two could be produced. As I show in this paper, many eminent botanists and gardeners, including Charles Darwin, both experimented with Adam’s laburnum and argued over how it might have been produced and what light, if any, it shed on the laws of heredity. Despite Jean-Louis Adam’s position and status as a nurseryman active within the Parisian plant trade, a surprising degree of doubt and scepticism was attached to his testimony on how the tree had been produced in his nursery. This doubt, I argue, helps us to trace the complex negotiations of authority that constituted debates over plant heredity in the early 19th century and that were introduced with a new generation of gardening and horticultural periodicals.

## Introduction

In 1830, the newly-established *Société d’Horticulture de Paris* announced in its journal that a local nurseryman, Jean-Louis Adam (1777–1830), had produced a new variety of cytisus. The tree, given the name *Cytisus adami* at the time but commonly referred to in Britain as Adam’s laburnum, produced both yellow and purple flowers. No cytisus being cultivated at the time had such striking properties. But perhaps more remarkable than the tree itself was Jean-Louis Adam’s testimony that he had not produced the tree by cross-pollination. He claimed instead that it was the product of a graft (Prevost and Poiteau 1830, 96). To the reading public of the *Annales de la Société d’Horticulture de Paris*, the claim had significant consequences: the editors of the *Annales* were suggesting that it was possible to produce plants then speculatively referred to as ‘graft hybrids’, a variety or even new species that was produced by the intermingling of the plant tissues of the root and the scion in a graft (Prevost and Poiteau 1830, 95). Graft hybrids represented a potential control over nature that held significant commercial promise, and they were also a much sought-after potential source of evidence in debates over the mechanisms of heredity. Adam’s laburnum was destined for commercial and scientific fame.

But while the tree became widely cultivated throughout Europe, and Adam’s testimony frequently repeated and defended in gardening and horticultural publications, few seemed to really believe Adam’s version of events.^1^ Even at the close of the 19th century, significant doubt remained attached to Adam’s testimony. On December 27th 1888, George John Romanes wrote to William Thistleton-Dyer, the assistant director at Kew Gardens, requesting help with an experiment.^2^ Romanes explained in a postscript:

I forgot to ask you if there would be any facilities in spring at Kew for repeating Adam’s graft of purple on yellow laburnum. I want to try this experiment in budding on a large scale because of its importance on Weismannism, should the result of any of the grafts go to corroborate Adam’s account of the way in which he produced the hybrid (Romanes 1896, 213).

Romanes’ idea for a ‘large scale experiment’ was far from new. New varieties could be propagated by grafting, but examples of new varieties that *resulted* from the act of grafting were very rare, the only historical example known in the 19th century being the ‘Bizarria’ orange (discussed below). The goal of Romanes and his predecessors was the same: to test the claims of the nurseryman Adam and his remarkable graft hybrid. Disagreement existed over the details of its creation. Adam had maintained in an interview that the tree was produced by a graft, and was not a sexually produced hybrid (Prevost and Poiteau 1830, 95–96). During the 19th century, the possibility of producing hybrids by grafting was hotly contested, and Adam’s testimony was one of the few sources of potential evidence in support of the idea. But as the botanist Robert Caspary declared in 1866, few people had ever believed Adam’s testimony (Caspary 1866, 849). This is surprising because, while effectively unknown to the scientific elite, Adam’s nursery was situated in Paris. Parisian nurserymen and seed companies wielded very strong authority within commercial, botanical, and horticultural spheres (Easterby-Smith 2015, 184). Seed companies like the family-owned Vilmorin Company worked to improve cultivated varieties, and the published methods tested and employed in commercial pursuits were read by Darwin and Galton (1998, 248). Nurserymen retained most of the authority on the practice of grafting, and it was common for authorities placed in eminent positions within French and British scientific networks to appeal to the authority of nurserymen and gardeners who were otherwise unknown. Well into the first decades of the 19th century, grafting was still given “the status of a liberal art as well as that of an empirical science” (Pacini 2010, 5). But Adam had institutional backing (of a kind) as well: his testimony concerning the tree was personally documented and published by the botanist Pierre-Antoine Poiteau (1766–1854), the head gardener of Fontainebleau and the editor of *Revue Horticole* (Oghina-Pavic 2015, 38). Gauging from contemporary reports, the tree became popular within nurseries throughout Europe, owing to its unusual characteristics (Caspary 1866, 849; Poiteau 1838, 6). Many who took an interest in Adam’s testimony were able to find a specimen to cultivate for themselves. So why was Adam doubted for most of the 19th century? Why was there a perpetual need for more proof, when most authorities who took an interest in the tree were happy to concede Adam’s honesty and professional status? Why did it remain what Darwin called ‘a strange puzzle’, despite its pedigree and explanation?^3^

Adam was a nurseryman in Paris; this brought with it a good deal of practical authority and at the same time suspicion of his abilities. Nurserymen and seed companies were integral to the identification of varieties and species that they sold; but so too were they the point at which many species and varieties were misidentified (Dewis 2014, 119). Was Adam a skilled practitioner who had relied upon his expert eye to spot the unusual branch, or had he made a more fundamental mistake in identifying his own stock? At the time that Adam first produced the laburnum and gave his testimony, the *Société d’Horticulture de Paris* had been established and had launched its own *Annales.* Although Adam was not a member of the *Société*, nurserymen were highly influential, contributing to new periodicals like the *Annales.* In France and in Britain, gardening and horticulture periodicals were creating new knowledge communities of readers and contributors. Within this new court of anonymous readers and contributors, proving or disproving Adam’s testimony became a point upon which reputations could be advanced or challenged. While many such anecdotes were deployed in debates over heredity, Adam’s testimony was held in doubt seemingly *because* the specimen itself was so available to scrutiny and study. Anyone with access to the plant might find a means of proving or disproving Adam’s story of how the tree was created, and this caused a problem for emerging sources of authority within the gardening and botanical sphere. This paper argues that Adam’s laburnum helps us to understand how the gardening and horticultural periodicals of the 19th century began to compromise the authority that nurserymen held on grafting and plant breeding. Specifically, it shows us how cultivating and observing specimens like Adam’s laburnum provided a means by which anecdotal stories like Adam’s testimony could be retold at emerging levels of scientific authority. By respectfully doubting Adam’s authority as a competent practitioner, Adam’s story of the tree’s origins could be re-told from a position of higher authority.

While the only records we have of Adam stem from interviews and investigations of his nursery by members of the *Société,* his position as a nurseryman active within Paris tells us a good deal about why his testimony and his own opinion of the plant’s origin mattered to botanists like Poiteau. For several decades, historians of biology and botany have worked to draw attention to the importance of the experiments and the labour of figures like Adam. Anne Secord was the first to suggest that the history of botany could be told from the perspective of labourers and artisans who contributed to its work (Secord 1994, 270). Gardeners and practitioners have long played a constitutive role in the development of plant heredity and plant sciences: not only conceiving of themselves as experimenters and part of a scientific discourse, but also in undertaking experiments and manipulations that fell outside the interests and terrain of institutional figures (Curry 2014, 542; Ratcliff 2007; Secord 1994; Spary 2003). Marsha Richmond, in her (2006) demonstrated that William Bateson’s Mendelian research relied heavily upon domestic environments. Helen Anne Curry has given what she terms amateur activity an important position within the history of biology, arguing that we need to attach more consequence to the practical desire on the part of plant breeders and practitioners stretching back into the 19th century to harness the powers in spontaneous variation (Curry 2014, 2016).

Looking at the long 18th century, Marc Ratcliff has argued that florists, cultivators, and livestock farmers, “understood their practices of transforming sorts and species [as] the influence of human art on nature” (Ratcliff 2007, 229). Both points, that gardeners (including nurserymen) played a key role in the experimental work, and also that they saw themselves as practitioners of a ‘human art’ upon nature, are important, as many authorities were quick to represent the experimental contributions of figures like Adam as wholly accidental or as the product of chance. Commercial nurseries in 18th century Paris, according to Easterby-Smith, enjoyed “patronage from an important network of scholars and wealthy collectors,” a dependency that remained true in 19th century France and Britain as well (Easterby-Smith 2015, 184). Ratcliff has shown that practitioners like Adam fit into hierarchies of authority, with practitioners commercially bound to the study and production of new varieties, while botanists remained politically and theologically dedicated to insisting on the fixity of species (2007, 211–212). Antoine Nicolas Duchesne (1747–1827), a practitioner who experimented on variation in Versailles, was a personal friend of many botanists in France and corresponded with botanists in Bern, Bologna, and Turin (Ratcliff 2007, 212). Adam seems to have been a peripheral figure in this world prior to 1830, and as I show below, Adam’s status as a nurseryman was put forward both as a means of trust and grounds for scepticism.

Much of the recent historiography on these communities consists of research focused on the colonial plant trade, where nurserymen played a key and integral part. This research has shown the influence and power wielded by nurserymen in Britain and France, particularly during the late 18th century.^4^ Sarah Easterby-Smith has argued that nurserymen and plant traders wielded influence over the scientific and commercial aspects of this sphere, first by marketing the science to a wider audience of amateurs, and secondly by being the principal agents behind the communication and diffusion of exotic specimens (Easterby-Smith 2013, 2015). McAleer, meanwhile, has looked at the significant role played by gardeners and nurserymen in colonial India and South Africa in maintaining the botanical networks that their European counterparts depended upon (McAleer 2016). Firms like James Veitch and Sons financed the collecting of exotic specimens abroad for its own nursery and also illustrations for *Curtis’ Botanical Magazine* (Olby 2000, 1044). Nurseries and the plant trade were essential to botany. While Adam was not known for his activity in these networks trading exotic plants, these networks nonetheless help us to understand the kind of authority and position that Adam wielded within botany as a plant trader active in Paris.

Within the historiography on the history of heredity, gardening has recently received more attention as a critical site for the production of evidence. At the start of the 19th century, as Carlos Lopez-Beltran has argued, the significance of the word ‘heredity’ had not yet come into widespread use in Britain (Lopez-Beltran 2004, 2007). But that did not mean that there wasn’t already a variety of ideas about the laws of generation, shared by medical, scientific, and lay people.^5^ Müller-Wille and Rheinberger attribute the distinction between ‘constant’ characters and ‘peculiarities’ of plants that botanists began to introduce to efforts by gardeners across Europe to acclimatise exotic and foreign plants to new climates (Müller-Wille and Rheinberger 2012, p. 14). This process was, they argue, constitutive of new interest in the idea that physiological characteristics are inherited by offspring from their parents. The belief that children, calves, and colts would grow to resemble their parents in both mental and physical attributes was assumed knowledge. “Like produces like,” is a phrase that populates the encyclopaedias, medical treatises, breeding manuals, gardening magazines of the first decades of the 19th century (and thereafter). Grand theories about heredity, like the inheritance of acquired characteristics, were founded on long lists of such anecdotes and cases that were gathered from personal observation, agricultural magazines, encyclopaedias, travel writing, and medical journals.^6^ Thomas Andrew Knight’s paper (Knight, 1837) on the inheritance of acquired habits in animals and plants, in addition to collecting many of the key stories that would inform later debates, also helped to establish a new form and genre for discussions of heredity and from 1839, the physiologist William Benjamin Carpenter included such lists of cases related to the inheritance of acquired characteristics in his *Principles of Physiology*, which he updated every 2 or 3 years with new cases (Carpenter 1839, 1841, 1851, 1854). But the production of evidence that mattered to debates over heredity only rarely began within the pages of an encyclopaedia or treatise: a lot of work to legitimate and grant authority to cases, and also to work out what kinds of *possibilities* the case represented in the first place, came before.

### The production of a puzzle

As Sarah Easterby-Smith has argued, French plant merchants in the late-18th century had helped to foster a strong public interest in botany and in gardening (Easterby-Smith 2013, 532–534). An extensive and affordable genre of botanical and horticultural books, filled with advice and instruction, as well as catalogues of their own plants and wares, lent a generation of *pépiniéristes* (nurserymen) a new authority and reach (Easterby-Smith 2013, 535). Among the many trends and fashions in cultivation, in the early decades of the 19th century, France and Britain were being invaded by numerous varieties of cytisus, a flowering tree that fit the new aesthetics of gardening that were transforming both public spaces and grand estates. Common laburnum (or *Cytisus laburnum*), the root stock that Jean-Louis Adam used, was particularly popular. “The *Cytisus Laburnum* is universally known,” Benjamin Maund declared in his catalogue of flowering trees (Maund 1825, 90). Describing one variety, Maund wrote: “Its slender decumbent branches, clothed, as the poet would say, with golden flowers and silver leaves, when lifted from their lowly birthplace, to the eminence their beauty deserves, are peculiarly attractive” (Maund 1836, 452). High commercial demand meant that, in Britain and France, nurseries were filling with numerous varieties of cytisus. Nurserymen propagated new trees by grafting, leading to nurseries that kept hundreds of trees as root stock from which new trees could be produced. Common laburnum was neither novel nor exotic to European markets. The end of the 18th century saw growing commercial interest in foreign and tropical plants (Arens 2015, 266; Curry 2012, 349–352; Richmond 2006). Illustrated catalogues like Maund’s were key to highlighting and encouraging consumer interest in varieties like common laburnum, but this also entailed that the familiar be repackaged and resold if it was to compete with the constant import of new, more expensive species from abroad (Dewis 2014, 41–43). Jean-Louis Adam kept hundreds of Cytisus laburnum in his nursery as root stock; a fact that speaks to his trust in the market for the variety, but a fact that also reveals how nurserymen were financially committed to the enduring popularity of certain plants. Experimentation with grafts and the production of hybrids by cross-pollination were an important part of the industry if only because the demand for novelty placed pressure upon nurseries (Elliott et al. 2011, 5, 168; Olby 2000).

The newly-established *Société d’Horticulture de Paris* (publishing its first prospectus in 1827) provided nurserymen and gardeners all across France with the opportunity to gain official membership with an institution that also provided a means of publishing and diffusing their methods, observations, and questions. Founded by a small group of wealthy amateurs, collectors, botanists and gardeners, the *Société* established a new centralisation of power within the horticultural and botanical world of France (Dubost 1994, 67). Like earlier societies established in Britain and France, *La Société d’Horticulture de Paris* hoped to establish a national interest in the scientific progress of horticulture. The *Société* saw itself as performing several roles: it would receive cuttings and seeds from its members in the hopes of establishing a collection, it would receive and publish letters from its members, and it would also play a didactic role in diffusing the teaching and ideas of its most important members (Anonymous 1827, 1–4). Like the Royal Horticultural Society (founded in 1804), the *Société* proposed to encourage scientific practice amongst the wider public, yet for many decades it served primarily the interest of a wealthy elite (Olby 2000, 1045). Importantly, Adam was not a member of the new society. From its first issue in 1827 to his death in 1830, Adam’s name only appeared in the *Annales* through Prevost’s and Poiteau’s communications. A mere 3 years’ after the establishment of the society, Adam’s position was that of an outsider, requiring interviewing and inspection by trusted members of the society before any discussion of the tree could proceed.

Because the tree was discovered by society members before Adam had any involvement in its popularization, it makes sense to introduce the first of three society members to seek out an interview with Adam, Nicholas-Joseph Prevost (1787–1855). One of the founding members, Prevost is described in the prospectus as a nurseryman in Rouen (Anonymous 1827, 32). Prevost was in fact, by 1830, an internationally recognised botanist and plant breeder, and one who maintained academic opinions: he was an adamant follower of the English botanist John Lindley’s (1799–1865) views on classification and the origin of varieties (Prevost 1829, v). The English landscape designer and publisher John Loudon’s (1783–1843) magazine had described him as “one of the most scientific nurserymen in France” (Loudon 1829, 372, 455). Prevost purchased, sometime in 1829, what he expected to be a purple-flowering cytisus from a fellow nurseryman in Rouen. Once it had flowered and displayed its unexpected mix of yellow and purple flowers, Prevost began the work of trying to trace back the origin of the tree. It took Prevost (by his own account) 1 year to trace it back to Adam, after two fellow nurserymen in Rouen helped him to establish the contact. A member of the *Soci*é*t*é, Prevost was quick to imply that Adam shared in a problematic ‘mercantile attitude’ within the gardening community (Prevost and Poiteau 1830, 93). Prevost enjoyed a scientific reputation as a nurseryman; but he enjoyed it in part by distinguishing the commercial-minded nurserymen as a breed apart. All that Prevost learned from his correspondence with Adam was that Adam had noticed a ‘vigorous branch’ very different from the others emerging from the graft. Prevost’s communication to the *Société* made it very clear that Adam himself was not of the mindset to understand or contribute to the understanding of the tree; but the editor, Pierre Antoine Poiteau, was not so quick to discount Adam’s usefulness.

Pierre-Antoine Poiteau published Prevost’s notice, but not before conducting his own visit to Vitry and seeking out Adam for an interview. Poiteau was a well-traveled botanist and gardener who had collected extensively in French colonies in the Caribbean and Africa. He served as head gardener at both Versailles and Fontainebleau, and served as *redacteur* for the *Annales* (Oghina-Pavic 2015, 38). Poiteau did not regularly investigate the reports of contributors to the *Annales* by personally visiting gardens and nurseries, but he had a special investment in the initial explanation that Adam had provided to Prevost. Since 1818, Poiteau had been interested in a tree known as the Bizzaria of Florence, or simply the ‘Bizzaria’. In his *Histoire Naturelle des Orangers,* lavishly illustrated by Poiteau himself, he depicted the fruit as half orange and half citron (Poiteau 1818, 107). As Poiteau and Antoine Risso explained in the book, the gardener who produced this tree claimed that it had first sprung from the spot of a failed graft on stock used for citrus trees (Poiteau 1818, 107–108). Poiteau, in 1818, was uncertain as to the veracity of the gardener’s story. Whether or not graft hybrids were possible, what interested Poiteau most was the possibility of several species existing *unblended* within one plant, where the “mixing” of qualities and characteristics that occurs in ordinary hybrids hasn’t taken place (Poiteau 1838, 7).

Poiteau shared Prevost’s view that Adam was not expected to contribute his own communication. He went to visit the nursery in Vitry himself. Poiteau wanted to see the root stock, but it had been already sold. Adam himself repeated that he had not seen the tree flower before selling it, but he told Poiteau that the branch had been taken from an eye that grew just at the point where the root stock and the scion were joined (Prevost and Poiteau 1830, 95). Neither Prevost nor Poiteau thought it remarkable that Adam should be able to recall the tree from 5 years’ earlier and such precise details even though he had not noticed anything extraordinary about it. Poiteau presented Adam’s innocence of the tree’s characteristics as validating the story: he suggested that Adam himself had no cause to dissemble or mislead anyone as to how he had created the tree (Prevost and Poiteau 1830, 96).

The key detail that Adam related to Poiteau was that the eye had grown from the joint of the graft and the scion. Still, Poiteau believed that another explanation might explain the tree’s existence: it might be that the root stock itself was produced from an “accidental development” (Prevost and Poiteau 1830, 96). He was split on the question as to whether or not to trust Adam’s account, as he would be for a decade. “I am far from sure,” he wrote, “that Mr. Adam is mistaken” (Prevost and Poiteau 1830, 96). He called for “amateurs” to experiment with the plant, and also suggested the name “*Cytisus Adami”* fixing the identity of the new variety or species firmly on the reputation of the nurseryman who had produced it (Prevost and Poiteau 1830, 96). The act of naming the plant was political in itself. As Emma Spary has argued, hybrids in the 18th century had held a dual value as monstrosities and also as “examples of man’s ability to transform Nature” (Spary 2000, 109). Poiteau himself remarked in his discussion of the Bizzaria in 1818 that the Academy did not designate monstrous fruits with particular names (Poiteau 1818, 108). He was arguably cautious in putting forward a definitive view because the distinction between species and variety was hotly contested in the 1820s and the 1830s. By allowing that Adam had potentially created something new, Poiteau was still allowing that Adam had demonstrated a new degree of power that mankind wielded over nature. His strongest endorsement of the possibility of graft hybrids came in 1838, when he revisited the relationship of Adam’s laburnum to the Bizzaria:

The gardener who first noticed the Bizzaria said that it was the product of a graft, but the scholar who published the discovery said that the Bizzaria had developed not by grafting, but from a wild orange tree. The scholar was believed, of course, and the gardener’s testimony taken for charlatanism, as is in order. This took place in Florence in 1644. However, the Cytise-Adam [Adam’s laburnum] might well rehabilitate the reputation of the gardener of Florence, and show that a graft can sometimes be modified so as to develop into a hybrid (Poiteau 1838, 6).

Poiteau’s renewed interest came from the discovery, after 1830, that the seeds of Adam’s laburnum could produce *either* common laburnum or purple cytisus. His belief in the possibility of unmixed species living within one plant was renewed. In defense of this idea he cited a further marvel—the existence of a cytisus “which has three species at once on the same root,” which he had observed in the nurseries of Versailles (Poiteau 1840, 59–60). Ordinary hybrids, Poiteau argued, partake equally of their parents. But this was no case of an ordinary hybrid, as the shoots on these plants demonstrated traits of separate varieties. Poiteau used the tree as an opportunity to put forward the idea that interested him most, that: “in the act of fecundation the fructifying substances have not mingled completely; that some particles have remained untouched; that they have lived and vegetated of themselves in the embryo and the seed” (Poiteau 1840, 60). He further promised that a M. Jacques of the Society of Horticulture had himself observed three distinct molecules in the sap of the Versailles cytisus. For whatever reason, nothing more was heard of this new cytisus; but as I show below, it was not the first grafting accident related to Adam’s laburnum to be left out of the discourse.

Visiting and corresponding with Adam had, by this time, become a clear means within the pages of the *Annales* of asserting one’s own reputation in the science of gardening, and so a third visit to Adam’s nursery occurred in 1833, when M. Camuzet undertook to visit Vitry himself. Camuzet was the head gardener of the Museum of Natural History in Paris; but by traveling to Vitry himself he gained the opportunity to publish his own account of Adam’s Laburnum in the *Annales*, and also to attack the vulnerable trust that Poiteau himself had invested in the story. Suspicious of the entire affair, Camuzet found that Adam had died, but that his nursery was still in business. Camuzet examined, by his own reckoning, a hundred specimens of cytisus that were being cultivated there by his family, in the hopes of understanding how Adam might have actually produced the plant. Camuzet’s opinion was that Prevost’s tree was nothing particularly special: Camuzet reasoned that Adam had misidentified his root stock, and that the eye that he saw growing from the graft was actually unrelated to the scion (Camuzet 1833, 197). The tree was an interesting hybrid in itself, but not a graft hybrid. Camuzet’s scepticism over Adam’s ability to distinguish varieties provided a means by which he could protect the authority and legitimacy of practicing gardeners like himself to decide on such matters: and while his account never gained the same influence of Poiteau’s, his communication remained part of the canonical texts that were gathering around the origin of the tree.

The expedition meant that Camuzet had a paper to deliver at the Society meeting on the 21st of August, 1833. Camuzet had two goals: to dispute the suggestion that there was a new species or variety that had been created, and to challenge the ability of Adam to understand what he was doing. Both contributed to his own reputation and authority, and helped to draw attention to himself as an overlooked authority on grafting. Camuzet charged that Adam was a good nurseryman, but that he did not have the eye of an observer (Camuzet 1833, 197). Casting doubt on Poiteau, Camuzet even maintained that he had found the root stock used by Adam in the nursery and that it was a hybrid itself, and not common laburnum (Camuzet 1833, 197). He was determined to cast doubt on the developing narrative. He reasoned that Adam’s laburnum was an ordinary hybrid produced by cross-pollination, and that the excitement around it had been an inevitable result from placing trust in the account that Adam made of his own practice. Good nurserymen and cultivators were not observers. Camuzet’s views cut no ice with Prevost or Poiteau; but the doubts he raised would linger for decades to come.

### Debate over Adam’s laburnum in *The Gardener’s Magazine*

The debate over graft hybrids was already a rich discourse in Britain before Adam’s testimony became widely known and debated (through the respective lenses of Poiteau and Prevost). As Pacini (2010, 1–2) showed in the case of 18th century France, grafting enjoyed the status of a science and an art, and this certainly held true in early-19th century Britain, where eminent authorities like Thomas Andrew Knight, president of the Royal Horticultural Society from 1811 until his death in 1837, had built his reputation on his knowledge and expertise in fruit tree grafts. But it was a skill shared by wealthy landowners, practitioners, gardeners, and common labourers, and Loudon’s lifelong ambition was to create a scientific community that could build upon this wide practice, potentially drawing upon the experiments and expertise of labourers and artisans. According to Sarah Dewis, Loudon worked to “refigure the gardener as a professional” (Dewis 2014, 33). The founder of the *Gardenesque* movement, Loudon held high sway within the world as both garden designer and publisher, before he had commenced with the publication of his magazine in 1826 (Simo 1981). Dewis views the nurserymen and gardeners who contributed to and consumed the magazine as receiving, for the first time, professional status where before they had ranked as labourers or artisans (Dewis 2014, 20). At the same time, Loudon sought contributions to his magazine from eminent botanists and gardeners.

Through his magazine, he maintained a dispute for authority with Knight. Loudon shared many of the same hopes and visions of the future of plant science that Knight held, but sought a different source of authority: practicing, professional gardeners. They disagreed on one crucial point. Knight had maintained since his first publications that there was no communication between the graft and the root stock of anything save nutrition (Knight 1795, 1801). Loudon not only discounted Knight as wielding little practical knowledge, but by his appeal to his readers for scientific observations of their own practices he actively sought to demonstrate that Knight was wrong, and that graft hybrids were feasible. The Royal Horticultural Society was founded in 1804 with a stated aim of providing a means of diffusing scientific practices to a passive audience. By the 1820s, publishers like Loudon were contesting this model. Loudon’s *The Gardener’s Magazine* aspired to create a community of scientific-minded gardeners who would contribute the results of their practice to the magazine, creating a more democratic space for the advancement of science (Dewis 2014, 52). This was not, however, a passive process on Loudon’s part. As Morris and Dewis have separately argued, Loudon’s model demanded an international roster of reliable gardeners and authorities who could contribute original communications (Morris 2004, 103; Dewis 2014, 53).

It is worthwhile to consider the treatment of cases related in detail to that of Adam’s laburnum that were discussed in the same period within *The Gardener’s Magazine.* In England, stories where grafting had effected some unexpected change in the plant were fairly common. Nurserymen were most often the source of such accounts, given their accumulation of practical experience. A nurseryman in London, George Bliss, published his manual on arboriculture in 1841, and *The Gardener’s Magazine* was quick to run a series of excerpts from the work. Among them was a significant account by Bliss of an experiment he had conducted in his youth:

I remember many years back, when quite a boy, a common white jasmine which was growing against the house, and being fond, even from my earliest years, of trying experiments among trees, I took a bud from a striped jasmine, and budded a branch on the green; the bud grew, and what shoots put forth below the bud, most of them became blotch-leaved: this is a proof the bud or graft must have an effect on the stock (Bliss 1842, 28).

No matter the confusion over what kind of entity was produced by grafting, the communication of peculiarities from scion to root spoke of a general principle: physical properties of plants, whether the result of sport or the influence of climate, could be directly transmitted.

William Falla, a nurseryman whose debts would drive him to suicide in 1836, maintained that grafts and buds could transmit their properties to the host tree (or stock). Having been selected to give an address to The Royal Horticultural Society in 1830, Falla contested Knight’s authority on the question by citing instead the case of ‘a gentleman’ in Herefordshire, “who, having a very old Golden Pippin apple tree which was in a dying state, planted around it several young seedling crabs, and, then they had established themselves, engrafted or inarched them into the trunk of the old tree; the consequence was, that in the course of a year or two, the old tree became nearly as healthy as ever it had been, from the vigour that was infused into it by the sap of the young crabs” (Falla 1828, 24). Falla, by his own estimation, had succeeded in transferring properties from one plant to another by means of a graft (Falla 1828, 24). His views were quickly reprinted by Loudon in his own magazine. Smocovitis (2009a, b) observes that these practices, well known by horticulturists and discussed in print since the 18th century, had a long history of documentation and debate. But one of the simplest instincts amongst both editors and gardeners were to seek more trials and observations. The editors here included a special encouragement to their readers to repeat the experiment, but unfortunately no later issue records any such experiment.

Commercial trade brought Adam’s laburnum to Britain faster than it did Adam’s testimony. Loudon presented Adam’s testimony as fresh news in the pages of *The Gardener’s Magazine* in 1839, but specimens of Adam’s laburnum had already been discussed and exchanged within the gardening and botanical circles of Britain. Loudon himself transmitted a cutting of a purple laburnum to Thomas Rivers in 1832, which he suspected was a cross between *C. Purpureus* and *C. Laburnum*, but there’s no mention in the notice of Adam (Rivers 1835, 24). Later in 1836, Thomas Rivers happened to go on a tour of France, which he documented for *The Gardener’s Magazine*. He happened to visit a ‘M. Camuset’ [sic], who demonstrated to him a hybrid that “came accidentally from seed among some common laburnums” (Rivers 1836, 224). It was Rivers’ first encounter with Adam’s laburnum, though he knew it had been “in some catalogues,” showing that by 1836 the variety was enjoying increasing trade (Rivers 1836, 224–225). It is interesting that Camuzet took this new opportunity to omit Adam’s role in the creation of the tree. The botanist William Herbert, whose 1837 *Amaryllidaceae* became a cornerstone text in plant physiology and hybridisation, included in the same work a short (but important) discussion of a tree belonging to his brother (Algernon Herbert), which he described as “a plant, grafted from a hybrid Cytisus, known to have been raised in France between *C. Laburnum* and *purpureus*” (Herbert 1837, 376). Herbert writes that after his brother had owned the plant for a year, a strong branch emerged from the root stock that bore all the characteristics of purple cytisus. The graft, Herbert maintained, had communicated its properties to the root. But Herbert was admittedly ignorant as to the provenance of the tree, knowing only that it had some celebrity in France. Nurseries and plant traders had been selling the tree for some time, but the name ‘*Cytisus Adami*’ or Adam’s laburnum had clearly not yet become widely used within the plant trade.

By 1838, Loudon included Adam’s laburnum in his enormous catalogue of trees in Britain (Loudon 1838), but without any mention of the tree’s origin. Only a year later did he included the story of the origin of Adam’s laburnum in the notices of his magazine (Loudon 1839, 122). Arguably, Loudon had known of Adam’s testimony for some time. Why did he not print it earlier? One possible reason was that despite his drive to build a science of horticulture upon the contributions and techniques of practitioners, he remained sceptical of Adam’s testimony. By 1839, *The Gardener’s Magazine* was decreasing its price to fight competition (Dewis 2014, 78), and his decision to publish on Adam’s laburnum might have been made in part out of desperation for content that would help the fortunes of his magazine. Engineering a dialogue on the tree for his magazine, Loudon sent the writings of Poiteau and Prevost to Herbert, and simultaneously wrote to Poiteau to request his current views on the tree. While he also encouraged gardener’s and readers to experiment with the tree and with graft hybrids, his choice of William Herbert is strong evidence that Loudon felt Adam’s word would hold little sway without an authority like Herbert to retell it. Unlike Poiteau, Herbert believed graft hybrids were a potential future source of improvement in horticulture and gardening. But his opinion on Adam’s laburnum was based on his own examinations of a live specimen.

Herbert’s letter to *The Gardener’s Magazine* retold the history of events: he criticized Prevost, partially agreed with Poiteau, and rejected Camuzet’s visit outright. Neither Prevost nor Camuzet enjoyed the same status as Poiteau, but Herbert explained carefully that his only reason “for giving credit to M. Adam’s assertion” was that he had personally seen plants grown from the seed of Adam’s laburnum which became more like common laburnum, or more like purple cytisus (Herbert 1840b, 383). In other words, Herbert maintained that he would have suspected that the tree could not have been an ordinary hybrid even had he not had access to Adam’s testimony. Herbert had already, based on his brother’s tree (presumably Adam’s laburnum), argued in 1837 that communication between grafts and rootstock could produce a “distinct plant” (Herbert 1837, 377). But Herbert had been (he claimed) ignorant of Adam’s testimony. Now, he strengthened his original position in his published reply to Loudon, asserting his belief that Adam’s laburnum “opens a field for the horticulturist to produce hybrid plants which perhaps could not be obtained by seed” (Herbert 1840a, 289). Adam’s laburnum, in Herbert’s opinion, had been created from a bud that grew on the seam between the tissue of the scion and root, allowing it to draw from both (Herbert 1840b). Herbert’s advice was just the kind that Loudon valued most: a new technique, discovered and diffused without the involvement of The Horticultural Society. Herbert rejected Camuzet’s scepticism, but he also departed from Poiteau’s views, establishing the view that would remain dominant in Britain: Adam’s laburnum was a distinct species or variety, a single plant that had been produced by the intermingling of common laburnum and purple cytisus. Rejecting Poiteau’s explanation that the plant was somehow home to a plurality of ‘distinct species’, Herbert recommended that cultivators could try to force the creation of similar buds by shield-budding and then killing the bud on the graft, in the hopes that a similar formation of hybrid buds would form at the site of the wound. Herbert’s reasoning was that the two different kinds of shoot that grow from Adam’s laburnum are, in fact, markedly different from their parents, and thus he reasoned that the plant as a whole was a new variety, a distinct individual from either of the two plants from which its life began.

Herbert provided an important endorsement of Adam’s claims, but he also stopped short of openly placing any real confidence or trust in Adam, and he wholly discounted the ideas of the nurseryman Prevost and the gardener Camuzet. The question of Adam’s laburnum, for Herbert, was one that could only be settled by investigation into the specimen’s fertility: and he strongly implied that the networks of trust established by nurserymen and gardeners were not a useful source of information in deciding how the tree had come about or by what means. While John Loudon encouraged experiment on the part of his readers, there were no further reports in *The Gardener’s Magazine* of Adam’s laburnum. In a small but significant way, William Herbert had contributed to the loss of authority held by nurserymen and gardeners on grafting. This also informs us of Loudon’s own ideas about the hierarchy of authority: while he extolled the virtues of collecting and diffusing the methods of gardeners lower on the professional ladder, the validity of their claims lay in the hands of more eminent figures.

### Darwin and Adam’s laburnum

By the time Darwin began to cultivate Adam’s laburnum in 1847, 17 years of debate and observation of the tree had taken place. His own thoughts on the plant deviated little from the positions put forward already by Herbert, although he sought (in vain) to rally more evidence from tissue samples, pollen, and experiments in cross-pollination. As I will show, Darwin corresponded with numerous botanists, seeking to understand not merely what Adam’s laburnum was, but whether there was support among the botanists he respected most for Adam’s version of events. Darwin himself explained in 1868 that: “the statement that *Cytisus adami* originated as a graft hybrid is so precise that it can hardly be rejected” (Darwin 1868, 396). Darwin failed to mention that he had all but rejected the statement for 20 years of experimentation and correspondence, and that he rejected many other statements by nurserymen and gardeners of similar results. He found division, particularly from Hooker, who remained sceptical of the entire business, but from other figures, particularly Robert Caspary in Germany, Darwin became increasingly convinced that Adam’s laburnum could be used as evidence for his developing ideas about heredity, finally positioning it as one of the key cases supporting pangenesis in *Variation* (1868). The importance of Adam’s laburnum to Darwin’s views on heredity has already been pointed out by Janet Browne (2003), and also by Olby (1985). Yongsheng Liu (2006) incorrectly credits Darwin with originating the notion of graft hybrids in the West, but nonetheless singles out the importance of Adam’s laburnum in developing Darwin’s views on plant heredity. Burkhardt, and later Secord and the editorial staff of *The Darwin Correspondence Project* have also highlighted and traced the importance of Adam’s laburnum to Darwin’s thought in their footnotes to his correspondence. Rather than focus on the importance of Adam’s laburnum to Darwin’s thinking, however, the correspondence in which he engaged provides a clear insight into how the puzzle over Adam’s trustworthiness remained impossible to unpick, despite the fact that an ever-increasing number of gardeners cultivated and experimented with the tree.

Darwin was cultivating Adam’s laburnum by June 1847, when he sends a clipping of what he describes as a ‘quasi-hybrid’ to Hooker (DCP-LETT-1094). In two separate letters sent in early June, Darwin expresses a keen degree of doubt: he wonders if botanists view individual petals and leaves as individuals, or if they tend to view a flower bud as a unit (DCP-LETT-1095). He was excited because his laburnum had sent up a raceme on which unexpected events were taking place. Amongst the yellow flowers, there seemed to be growing also a yellow flower whose calyx was half-purple. Hooker did not see what Darwin saw; he viewed the anomaly in the flower as arising from “irregular development,” and not as a feature of the plant’s normal growth (DCP-LETT-1097). The half-purple, half-yellow calyx wasn’t added to Darwin’s discussion of Adam’s laburnum in *Variation*; but neither was he converted to Hooker’s position. The idea that Adam’s laburnum was a mingling of two separate races, a mingling that had occurred through a graft, and which could be propagated by seed, reinforced Darwin’s belief that the entire body of the organism had a role to play in determining heredity. Hooker, however, remained of the opinion that Adam’s testimony was mistaken. Where Darwin saw evidence of a graft hybrid, Hooker only saw accidental characteristics.

In 1854, Darwin reminded Hooker of the incident of the flower on his laburnum that threatened to exhibit a mixture of blue and yellow petals; he maintained that he was confused over whether it was evidence of individuation in separate leaves and petals, or, as he would note later in *Variation*, the flower was exhibiting a tendency to reversion (DCP-LETT-1588). Still, he worried that Hooker doubted the significance of Adam’s laburnum to questions about plant heredity. Darwin wrote, “I cannot see how there well can be a fallacy, but then I am as credulous as you are sceptical,—oh, how credulous I must be!–” (DCP-LETT-1908). Darwin’s concern shows strong proof that the reputation that nurserymen like Adam had enjoyed earlier in the century had continued to erode. Darwin’s solution to his botanical credulity was to begin seeing for himself if he could prove the tree was the result of ordinary cross-pollination.

We don’t know how long these efforts continued, but in July 1862 Darwin mentions in a letter to Daniel Oliver: “*Cytisus adami* is a strange puzzle; I have failed in fertilising *C. purpureus* by pollen of common Laburnum” (DCP-LETT-3664). The failure to cross-pollinate the variety that served as the root for the scion of Adam’s laburnum bothered Darwin for the rest of the year; the notion that the ‘quasi-hybrid’ was a puzzle remained in his thinking. “That Laburnum case seems one of the strangest in physiology,” he wrote to Thomas Rivers later in the year. “I have now growing splendid, fertile yellow Laburnums (with long racemes like the so-called Waterer’s Laburnum) from the seed of yellow flowers on the *L. adami*” (DCP-LETT-3879). Like Herbert, Darwin felt that the fertility of Adam’s laburnum would provide the solution to trusting or rejecting Adam’s testimony. But the veracity of his testimony remained the purpose of his experimentation: like Loudon and Poiteau before him, Darwin was invested in the feasibility of graft hybrids for his emerging ideas about heredity and pangenesis.

Around this time a new case appeared. A nurseryman, E. Purser, wrote to John Lindley’s *The Gardener’s Chronicle* claiming that he had been able to repeat the production of a graft hybrid between purple cytisus and common laburnum:

Laburnum Sports.–I forward a specimen branch of a Laburnum now growing in my garden. Some few years since three grafts of the Cytisus were inserted, and now the whole character of the tree is changing, and every year since losing the yellow flower of the Laburnum, and producing the short purple flower, as you see. I am not aware whether it is an unusual occurrence, but large branches (quite removed from the Cytisus) produced flowers of both characters, and are now in full bloom (Purser 1857, 382).

Here was an ideal case: the scion affecting the root stock, confirming that his preferred interpretation of Adam’s Laburnum was correct. Lindley knew the value of the testimony to his readers. “No point in vegetable physiology is of greater interest to gardeners,” he wrote, “than the influence, if any, of the scion upon its stock; or vice versa” (Lindley 1857, 400). Lindley, much as Poiteau had almost two decades earlier, now put his own authority as editor into the task of legitimating Purser’s experiment. Referring back to Adam’s Laburnum, he wrote: “Here then are two notorious cases, the history of each perfectly ascertained […] we are fully justified in assuming that similar changes, greater or less, will inevitably attend the union of any other two plants. We must regard such changes as resulting from some constant law, although we may continually fail to perceive its presence” (Lindley 1857, 400). Lindley pushed that the two cases were now indications that “some constant law” was at work. But Lindley did not visit Purser. Darwin easily rejected Purser’s trustworthiness, writing of the claim that: “more evidence and copious details would be requisite to make so extraordinary a statement credible” (Darwin 1868, 389), even as he assumes a degree of veracity in Adam’s testimony.

As Smocovitis (2009a, 222) observes, the years of 1857 forward were ones where Darwin was immersed in compiling botanical cases. Smocovitis has argued that Darwin (1868a, 389) were dependent upon these schools of technical knowledge that trafficked amongst gardeners and plant breeders, and in the case of Purser’s laburnum, Darwin’s interest was sparked; but also his suspicion. In the next issue, Darwin wrote a query to Lindley. Could Purser discover if the seeds were sterile? Were the leaves changed as well as the flowers? Darwin recounted his success in raising pure yellow laburnums from the seeds of Adam’s Laburnum, and wondered if the idea of telegony—“as the physiologists have explained”, might be proven true (Darwin 1857, 421). But the conversation sparked other, murkier questions from the readers of the *Chronicle*. A gardener, Mr Madden in Ballinasloe in Ireland, wrote to say that he had observed yellow shoots on his purple laburnums several times (Madden 1857, 454). Another gardener, George Fitt in Norwich, wondered why his laburnum grew yellow flowers on the left side and purple on right (Fitt 1857, 421). Lindley’s *Gardener’s Chronicle* was reaching the limits of what it could authenticate. Unlike the treatment of Adam in France by the *Société d’Horticulture de Paris*, no one in Britain undertook to visit Purser, Fitt, or Madden. Lindley had no mechanism in place to sort out cases of ‘irregular development’ from those of genuine interest. And every triumph of cultivation that seemed to evidence the ability of the root to inform the scion inevitably produced a series of queries and letters that raised more questions and problems than did the example answer.

Despite Lindley’s belief that he had successfully discovered and communicated a second incident of Adam’s Laburnum, and Darwin’s welcome suggestion that perhaps more such cases had now been observed, in his 1868 discussion Darwin rejected Purser’s case as untrustworthy while maintaining a circumspect trust in Adam’s. The testimony of gardeners in publications like *The Gardener’s Chronicle* could be rallied when it supported the main bent of theory, but it could also be easily ignored when it seemed improbable or disagreed. It took far less work to dismiss a gardener who contributed to the Chronicle than it did to legitimate and lend authority to the anecdotal cases submitted to the magazine.

Still, by the 1860s, Darwin had decided that his opinion aligned with that of Lindley: there was a constant law at work, and Adam’s Laburnum was evidence of its operation, even if Purser’s Laburnums failed to convince him. Graft hybrids, he believed, were perhaps [a good source] of experimental evidence that could be obtained in support of his emerging notion of pangenesis (Endersby 2003, 81; Holterhoff 2014, 672), even if there were obvious challenges to repeating the fortunate accidents that had taken place in Vitry, and centuries earlier in the case of the Bizzaria. While his own laburnum died in 1866, part of Darwin’s renewed faith came from his correspondence with Robert Caspary. Like many others, Darwin’s interest in Adam’s laburnum was easily revived by the promise of new information. Caspary’s argument that Adam’s laburnum could not be an ordinary hybrid probably played a key role in Darwin’s decision to give the puzzling case pride of place in *Variation*. A good deal of his discussion of the tree ended up coming from Caspary’s pamphlet and correspondence.

In 1865, during his tenure as a botanist at Konigsburg, Caspary believed he witnessed root stock communicate properties to a graft in a case where a white moss-rose had been grafted onto a cabbage rose (DCP-LETT-5018). The observation prompted Caspary to revisit what he claimed were established cases such as Adam’s laburnum in an address to the Amsterdam-congress. Darwin read of the congress in Lindley’s *The Gardener’s Chronicle* (DCP-LETT-5018). The authority of Caspary seems to have been all that Darwin finally needed in order to embrace what he had referred to a decade earlier as his credulity: Variation would put forward Adam’s Laburnum as strong evidence in favour of the capacity of the root stock to inform the properties of a graft, and also of the physiological processes that Darwin was now referring to as ‘pangenesis’. After reading Caspary’s paper, Darwin wrote to Thomas Rivers for seeds in order to observe for himself some of Caspary’s claims that Adam’s laburnum was no ordinary hybrid:

…Why I want them is that Prof. Caspary states that in C. Adami the pollen in appearance is good, whilst the ovules are bad. Now this does not occur in any known sterile ordinary hybrid, & Caspary hence argues that C. Adami is not a common hybrid (DCP-LETT-5070).

After viewing the sample for himself, Darwin decided that he agreed with Caspary on the question of the pollen: he thought it looked monstrous (Darwin 1868a, 388). The fact helped Darwin dismiss the long-running argument that Adam’s Laburnum had been produced by cross-pollination. Darwin always viewed graft hybrids—if they could be proven to exist—as a key and vital piece of evidence in support of pangenesis:

It was shown in the discussion on graft-hybrids that there is some reason to believe that portions of cellular tissue taken from distinct plants become so intimately united, as afterwards occasionally to produce crossed or hybridised buds. If this fact were fully established, it would, by the aid of our hypothesis, connect gemmation and sexual reproduction in the closest manner (Darwin 1868a, 387–388).

Darwin’s later reliance on the case was noted by his contemporaries in their reviews of the arguments put forward in *Variations*. An anonymous reviewer highlighted his reliance on Adam’s laburnum in the *Westminster Review* (Anon, 1868), and Alfred Bennett (1871) would later claim in the pages of Nature that Darwin’s argument for pangenesis rested in part on the evidence provided by Adam’s laburnum. The importance of the example of Adam’s Laburnum to Darwin’s argument wasn’t lost on Alphonse de Candolle, who wrote on the 2nd of July 1868 to say that he wasn’t particularly convinced by Caspary’s arguments or *those of others* who claimed there was something special about Adam’s Laburnum (DCP-LETT-6264). But the 1870s, a period that saw numerous experiments hoping to demonstrate pangenesis through grafting and budding, would maintain widespread optimism that Adam’s laburnum was a genuine graft hybrid, and that a future method of producing such hybrids lay within reach.

Still, Adam’s testimony was, in Darwin’s final estimation, valid because of Adam’s professional standing. It was an ironic end to several decades of experimental work, that despite years of investigating the properties of Adam’s laburnum, Darwin’s response to sceptics could only appeal to Adam’s trustworthiness: “On the other hand, we have a clear and distinct account given by M. Adam, who raised the plant, to Poiteau” (Darwin 1868, 390). Thirty years of efforts by various botanists to provide an experimental proof of Adam’s claim had not produced a means by which trust in the authority of nurserymen was dispensable. In the final analysis, the best evidence for graft hybrids remained the clear and distinct account given by Adam himself, despite the protracted contest to either improve upon or refute his testimony.

## Conclusion

As I have demonstrated, Adam’s position as a nurseryman was both the means by which his extraordinary claims were justified and also the grounds for calling his testimony into doubt. Grafting, as a science and art in its own right, had long been a practice that fell within the authority of nurserymen, gardeners, and plant traders. But with the emergence of a new generation of gardening periodicals and institutions in the early decades of the 19th century, this authority was compromised, particularly from academic and eminent sites of authority. Because Adam’s story was inextricably linked to a live specimen, a dialogue emerged wherein numerous authorities sought to reinforce their own expertise by vindicating or refuting Adam’s testimony with their own observations. These authorities sought to carefully balance their scepticism of Adam with respect for the integral role that nurserymen played within the plant trade, upon which academic and scientific authorities were wholly dependent. Eventually, the commercial success and popularity of the tree no doubt played some part in the eventual acceptance of Adam’s testimony. Rather than encouraging a generation of experimentation with graft hybrids, most of these authorities focused solely on investigating specimens of Adam’s laburnum: it was only nurserymen and unknown gardeners who seem to have carried on the experimental work of trying to reproduce Jean-Louis Adam’s graft. This provides further evidence that the real puzzle facing authorities was not the tree itself, but the means by which Adam’s testimony could be verified within the anonymous networks emerging with the periodical trade.

Adam’s own testimony about how the tree was created found new support in the 20th century. The idea that Adam’s laburnum was a chimera, or a plant composed of two different somatic tissues growing side by side, was put forward with experimental backing in 1907 by Hans Winkler, yet even then discussion and debate continued for decades as to the nature of the numerous cultivated plants that fell under the heading of ‘chimera’ (Cowles 1911, 148; van Harlen 1998, 262). But the puzzle over Adam’s laburnum had not really focused on its physiological properties in the 19th century. Instead, as I have argued, the puzzle was over the trustworthiness of Adam himself. The tree appeared to hold the secret to any number of pressing questions about generation, heredity, plant breeding and crop improvement: but many of the most important questions rested on the testimony of a nurseryman who was largely unknown to the emerging societies and institutions that took plant breeding and cultivation as their object of study.

Despite the reputation that Parisian nurserymen enjoyed in the early decades of the 19th century and their crucial role in establishing horticulture and botany as domains of science open to amateurs and aristocrats, Adam’s laburnum shows us that the authority wielded by nurserymen became increasingly constrained and difficult to negotiate as the century progressed. This was in part because the very periodicals that we often understand as providing practitioners with a scientific platform were in fact at the same time compromising their control over the idea of expertise and observation. Darwin’s own insistence in 1868 that Adam’s testimony was precise and trustworthy was insincere, and concealed decades that he had spent attempting to arrive at some means of providing a means of trust that the botanical community would recognise and accept. But the work of gardeners and nurserymen like Purser, Madden, or Fitt, who all communicated interesting results to *The Gardener’s Chronicle* did not seem to provide the basis for this means: Darwin never corresponded with any of these figures, and the efforts to investigate Jean-Louis Adam in 1830 were noticeably absent by 1857.

Despite the fact that seeds, cuttings, pollen, leaves, and preserved flowers were traded between authorities and institutions, it was difficult for figures like Darwin, Caspary, and Hooker to agree on what they *saw*. As Anne Secord has demonstrated, botanists shared a convention of examining specimens that was established through the production of illustrated botanical works (Secord 2011). However, disagreement over colouration, over deformity, and over resemblance meant that many of the letters exchanged between Darwin and other botanists were not decisive or convincing for the recipient. Few changed their minds.

Despite these tensions, the industry that individuals and editors put into diffusing and promoting Adam’s laburnum kept interest and debate over the possibility of producing graft hybrids alive through key decades of experimentation and research. The promise of Adam’s laburnum that graft hybrids were a possibility, and the larger possibility that pangenesis was a theory of heredity that had already amassed experimental evidence in its favour from graft hybrid cases like it, informed and shaped the lay of the land in the plant sciences and in the emerging science of heredity.

From the first ‘discovery’ of the tree by Prevost, gardeners, botanists, and finally evolutionists (like Romanes) sought to use the specimen to bolster their own reputations as experts within the discourse on graft hybrids. With every decade of activity around the tree, the failure to replicate Jean-Louis Adam’s graft raised the problematic question of how, if there really was a ‘constant law’ at work behind the creation of the tree, that law was so difficult to demonstrate a second time. Gardening magazines remained home to excited letters explaining the creation of graft hybrids like Adam’s laburnum throughout the 19th century, and Darwin was by no means the only eminent authority compiling these cases for future use. Adam’s laburnum was a puzzle in itself, but more significantly it served as a puzzle piece within the wider mosaic of heredity, that Darwin and others were attempting to assemble from hundreds of such cases. Their individual legitimacy was important from the outset, as piece by piece, they formed what, in 1888, Romanes clearly still believed to be the forms of evidence that would make or break the theoretical understanding of heredity of his day.

## Footnotes

1 See Caspary (1866, 849).

2 This letter was sent during a heated exchange between Romanes and Thistleton-Dyer in the pages of *Nature*. See Forsdyke (2001, 224–226).

3 See Darwin’s correspondence with the American botanist Thomas Rivers. Darwin, C., to Rivers, T. 28 December 1862. (DCP-LETT-3879). Accessed 18 April 2017. All references to Darwin's correspondence, except where otherwise noted, are taken from the Darwin Correspondence Project and identified by the DCP Letter number.

4 As John McAleer puts it, “focusing on the movement of plants—as well as the institutions established to succour, store, and study them—offers ways of interrogating the relationship between the applications of science and the consolidation of empire” (McAleer 2016, 25). Emma Spary argues that there was substantial investment on the part of aristocratic and political powers in France throughout the 18^th^ century interested in the powers of exotic plants, a fact which placed practitioners with technical knowledge in positions of new influence (2004, 18).

5 The importance of multiple agents to the emerging science of heredity has received strong attention recently. Wood (2007) has studied the importance of 18th century view on sheep-breeding, while Ayres (2009) has expanded our understanding of how debates over bud variation in the 18th and early 19th century informed wider ideas about generation and inheritance.

6 See for instance Jenkins (2015) on the importance played by lists of anecdotal evidence in early-19th century debates over heredity.
